# Increased incidence of sexually transmitted diseases in the recent years: data from the ICONA cohort

**DOI:** 10.7448/IAS.17.4.19653

**Published:** 2014-11-02

**Authors:** Antonella Cingolani, Stefano Zona, Enrico Girardi, Alessandro Cozzi Lepri, Laura Monno, Eugenia Quiros Roldan, Giovanni Guaraldi, Andrea Antinori, Antonella d'Arminio Monforte, Simone Marcotullio, on behalf of ICONA Foundation Study

**Affiliations:** 1 Division of Infectious Diseases, Department of Public Health, Catholic University, Rome, Italy; 2 Division of Infectious Diseases, University of Modena and Reggio Emilia, Modena, Italy; 3 Department of Epidemiology, National Institute for Infectious Diseases, Rome, Italy; 4 Infection Population Health, University College London, London, UK; 5 Division of Infectious Diseases, University of Bari, Bari, Italy; 6 Division of Infectious Diseases, University of Brescia, Brescia, Italy; 7 Clinical Department, National Institute for Infectious Diseases, Rome, Italy; 8 Health Sciences, San Paolo Hospital, University of Milano, Milano, Italy; 9 Nadir Foundation Onlus, Rome, Italy

## Abstract

**Introduction:**

Sexually transmitted diseases (STDs) data collected in HIV+ patients could be used as indicator of risky sexual behaviour possibly linked to HIV transmission. We described the STDs incidence over time and identified higher incidence factors.

**Methodology:**

All patients in the ICONA Foundation Study enrolled after 1998 were included. STDs considered: any-stage syphilis, human papilloma virus (HPV) diseases, gonococcal and non-gonococcal urethritis, herpes simplex virus (HSV) genital ulcers, vaginitis and acute hepatitis B virus (HBV), hepatitis A virus (HAV), and hepatitis C virus (HCV) infections (only for non-IVDU (intravenous drug user) patients). STDs incidence rate (IR): number of STDs divided by person years of follow-up (PYFU). Calendar periods: 1998–2002, 2003–2007 and 2008–2012. Predictors of STDs occurrence were identified using Poisson regression and sandwich estimates for the standard errors were used for multiple STD events.

**Results:**

Data of 9,168 patients were analyzed (median age 37.3 (SD=9.3), 74% male, 30% MSM). Over 46,736 PYFU, 996 episodes of STDs were observed (crude IR 17.3/1,000 PYFU). Median (IQR) CD4/mmc and HIV-RNA/mL at STD: 433 (251–600) and 10,900 (200–63,000). Highest crude IRs were observed for any-stage syphilis (3.95, 95% CI 3.59–4.35), HPV diseases (1.96, 1.71–2.24) and acute hepatitis (1.72, 1.49–1.99). At multivariable analysis (variables of adjustment shown in Figure 1), age (IRR 0.82 per 10 years younger, 95% CI 0.77–0.89), MSM contacts (IRR 3.03, 95% CI 2.52–3.64 vs heterosexual) and calendar period (IRR 1.67, 95% CI 1.42–1.96, comparing 2008–2012 with 1998–2002) significantly increased the risk of acquiring STDs. Moreover, having a HIV-RNA >50 c/mL (IRR 1.44, 95% CI 1.19–1.74 vs HIV-RNA <50 c/mL) and current CD4+ cell count <100/mmc (IRR 4.66, 95% CI 3.69–5.89, p<0.001 vs CD4+ cell count >500) showed an increased risk of STDs. Being on ARV treatment significantly reduced the risk of developing an STD (IRR 0.37, 95% CI 0.32–0.43) compared to ART-naïve people, even in the situation of temporary interruption of treatment (IRR 0.51, 95% CI 0.39–0.43) (see Figure 1).

**Conclusions:**

The overall incidence of STDs has been increasing in the recent years. Interventions to prevent STDs and potential further spread of HIV infection should target the recently HIV diagnosed, the young population and MSM. Being on ARV treatment (potentially an indicator of whether a person is regularly seen for care) seems to reduce the risk of acquiring STDs independently of its viro-immunological effect.

**Figure 1:**
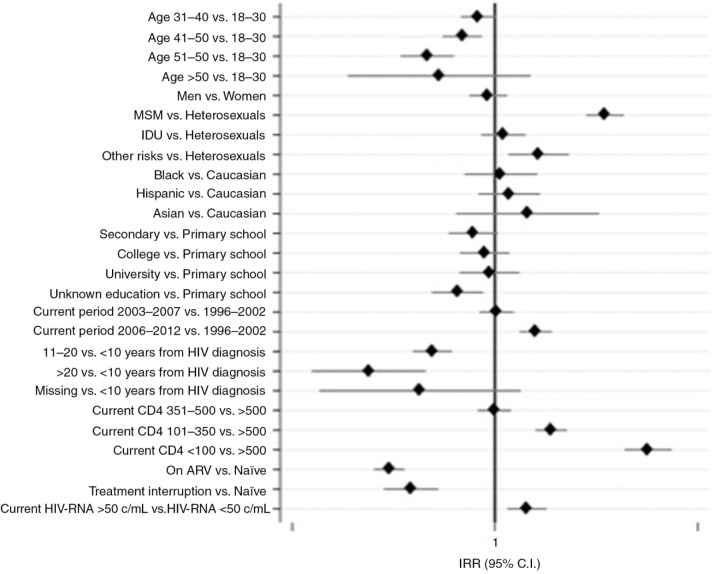
Predictors of acquiring STD at multivariable Poisson regression analysis.

